# Editorial: Circadian rhythm in cellular endocrinology

**DOI:** 10.3389/fendo.2024.1429793

**Published:** 2024-05-28

**Authors:** Anthony H. Tsang, José Cesar Rosa-Neto

**Affiliations:** ^1^ Institute of Metabolic Science-Metabolic Research Laboratories, University of Cambridge, Cambridge, United Kingdom; ^2^ Department of Cell and Developmental Biology, Immunometabolism Research Group, Institute of Biomedical Sciences, University of Sao Paulo (USP), Sao Paulo, Brazil

**Keywords:** circadian rhythm, endocrinology & metabolism, cardiovascular risk, stress responses, chronotherapy

The drastic fluctuations of environmental factors over the course of a day compelled organisms to evolve an intrinsic timekeeping mechanism – the so-called circadian clock – allowing for anticipation and adaptation to the daily predictable recurring changes.

The mammalian circadian clock is comprised of a distributed cellular timing system organized in a hierarchical manner. At the molecular level, cellular circadian rhythms are controlled by a genetically encoded machinery. This in turn is comprised of an interlocked transcriptional-translational feedback loop of a set of ubiquitously expressed clock genes which drive the rhythmic expression of a host of target genes, known as clock-controlled genes, in a tissue-specific manner. The suprachiasmatic nucleus (SCN) of the hypothalamus serves as the master clock that synchronizes the peripheral and other central nervous systems (CNS) clocks to each other and to the external light-dark cycle (1).

In mammals, endocrine systems are one of the major outlets of the circadian clock to regulate physiological processes. Emerging evidence shows that many hormonal systems are subject to circadian regulation – circulating levels of many endocrine factors as well as the responsiveness of target tissues to these factors oscillate over the 24-hr daily cycle (For a comprehensive review, see (1)). Among those, the glucocorticoids (GCs) and melatonin play a special role in circadian biology. Not only are their syntheses, releases, and target tissues’ sensitivity regulated by the circadian clock, GCs and melatonin also play a major role in synchronizing cellular clocks of a wide range of tissues in the CNS and the periphery (2, 3). More recently, other hormones such as adiponectin – an adipokine and oxyntomodulin – and incretin have also been found to reset cellular clocks, albeit in a tissue-specific manner (4, 5). Thus, not only do endocrine factors serve as mere outputs of the circadian systems, but they also feedback and contribute to the robustness of it at various levels.

Glucose homeostatic regulation by insulin illustrates one of the best characterized endocrine process regulated by the intricate interplays between central and peripheral clocks: the CNS clocks play a role in controlling energy balance and regulating whole-body insulin sensitivity (6), the pancreatic beta-cell clock has been shown to regulate insulin release (7), and circadian clocks in metabolic tissues fine-tune local insulin sensitivity (8). Misalignments of these clocks, such as those induced by mistimed food consumption of highly palatable energy-dense food, leads to a state of internal circadian desynchrony which has been established as a risk factor for obesity and related metabolic disorders (9). Several other hormonal systems have been found to be regulated by the circadian clock following similar principles.

In this Research Topic, we highlight recent progress in the understanding of the interaction between cellular circadian rhythms, endocrine functions, and physiological homeostasis. Various aspects of the cardiovascular system have long been shown to display diurnal and circadian rhythms. Notably, there are higher incidences of complications during the morning (10). Yu et al. summarized the current evidence of plasminogen-activator inhibitor 1 (PAI-1) – a secreted inhibitor of plasmin proteolytic activation (and hence a positive regulator for thrombosis) - as an important mediator for circadian influence of cardiovascular complications in a very interesting narrative review.

The stress response coordinates behavior and physiology to cope with imminent perceived dangers. Various components of the stress response have been shown to be subject to circadian regulation (11). Two original studies in this Research Topic provide new insights into this research area. Using a cell model of hypothalamic neurons, Alcántara-Alonso et al. have uncovered a molecular mechanism wherein a hypothalamic stress-induced neuropeptide urocortin 2 differentially regulates the expression of hypothalamic appetite-regulating neuropeptides and clock genes via engaging type 2 corticotropin releasing hormone receptor (CRH-R2), illustrating a potential mechanism via which the stress responses reset circadian behaviors. On the other hand, using a mouse model with selective deletion of a pleotropic protein deacetylase SIRT1 in cartilage as a model of growth impairment, Shtaif et al., reveal that cartilage SIRT1 plays an unexpected role in cognitive and anxiolytic function, providing a plausible link between body growth and cognitive development. Though this study does not observe circadian abnormities in these mutant mice under normal light-dark holding conditions, it remains interesting to explore the circadian behavior and cartilage biology under constant conditions, as SIRT1 has long been known to regulate clock gene oscillations in both the SCN master and peripheral clocks (12).

Furthermore, misaligned dietary timing has been extensively linked with several metabolic diseases. In this Research Topic, He et al., demonstrated in a mouse model of diet-induced cholesterol gallstone formation that improper timing of feeding exacerbates the disease progression, correlating with dysregulated cholesterol and hepatic bile acid metabolism as well as fluctuation in gut microbiota.

In summary, the circadian clock is extensively intertwined with endocrine functions, from the cellular to the interorgan levels. As a major output of the circadian clock, novel insights into elucidating the interaction between the circadian and the endocrine systems will help in developing therapeutic strategies to various forms of metabolic, endocrine, and circadian rhythm sleep disorders.

## Author contributions

AT: Writing – original draft, Writing – review & editing. JR-N: Writing – original draft, Writing – review & editing.
